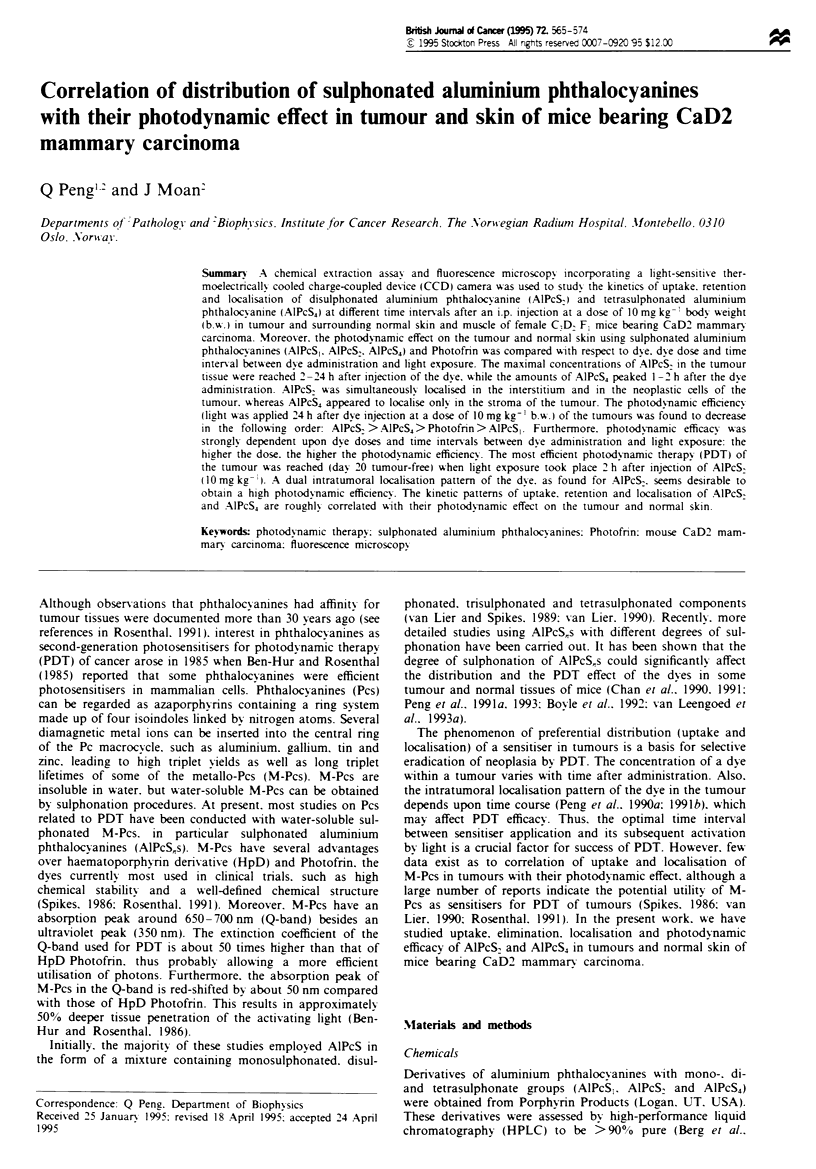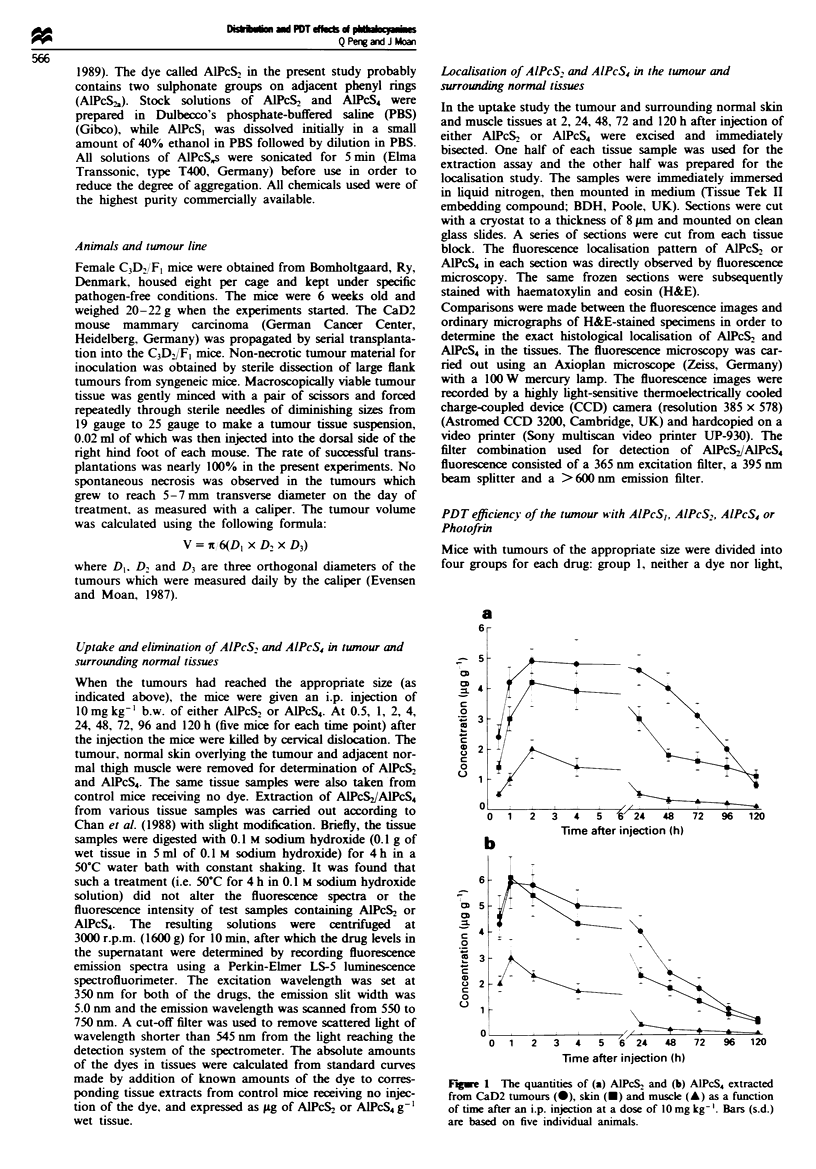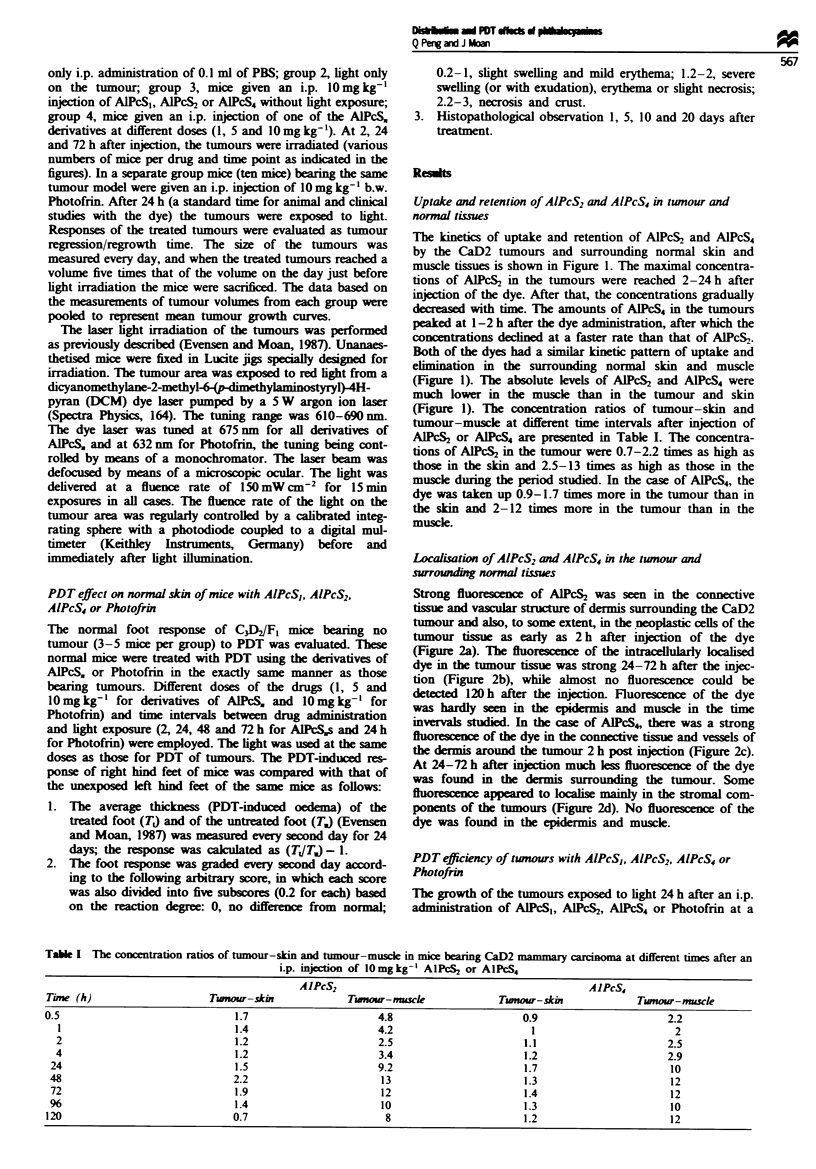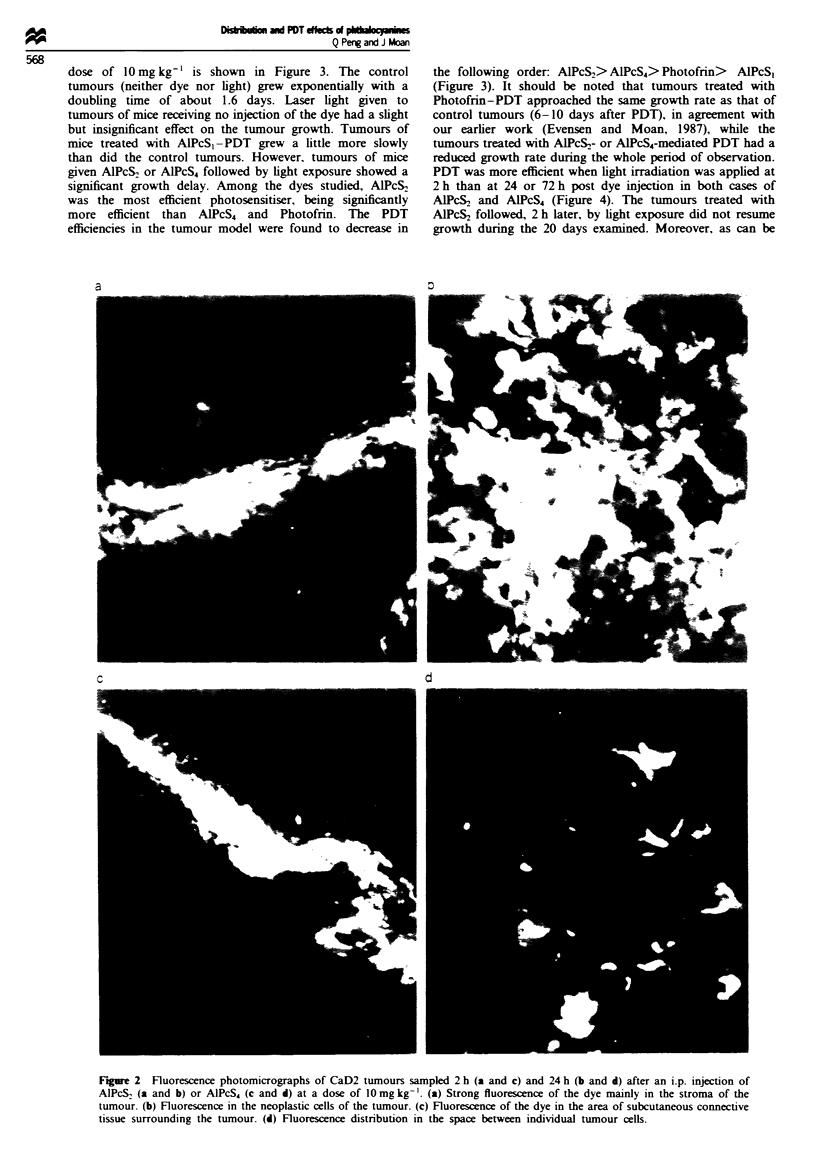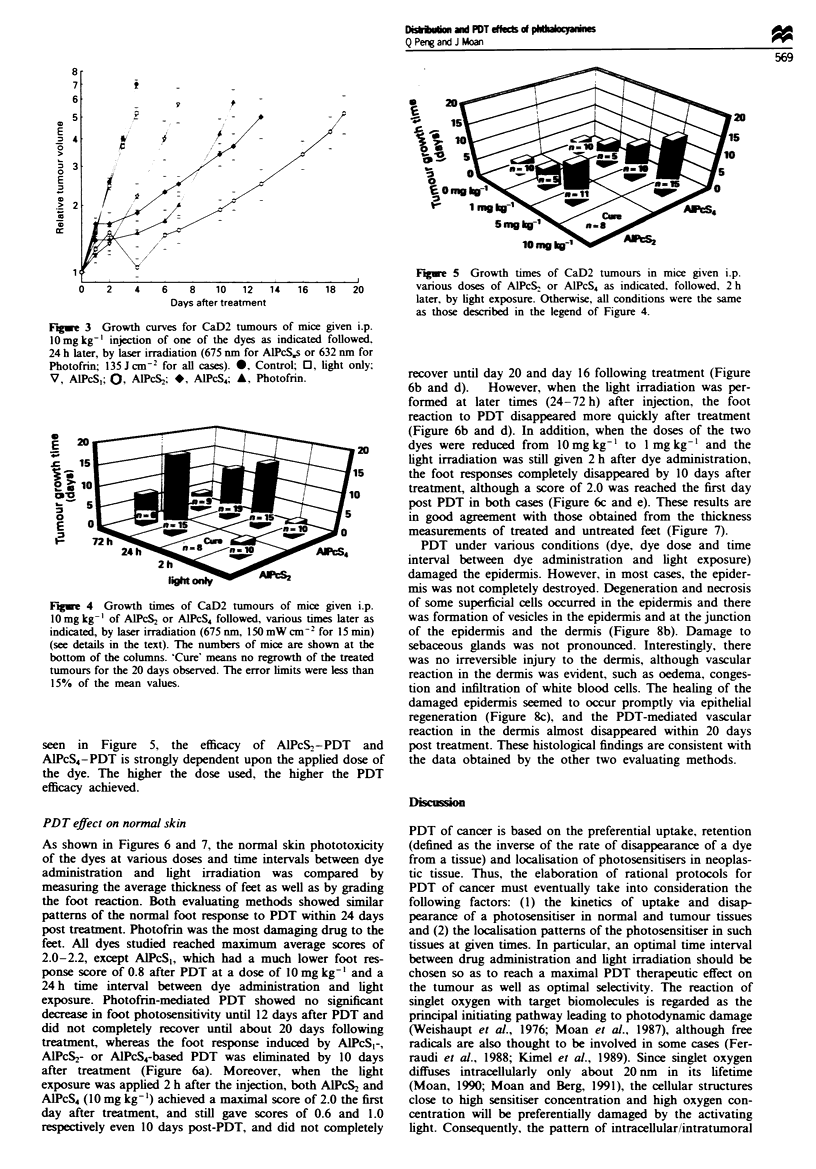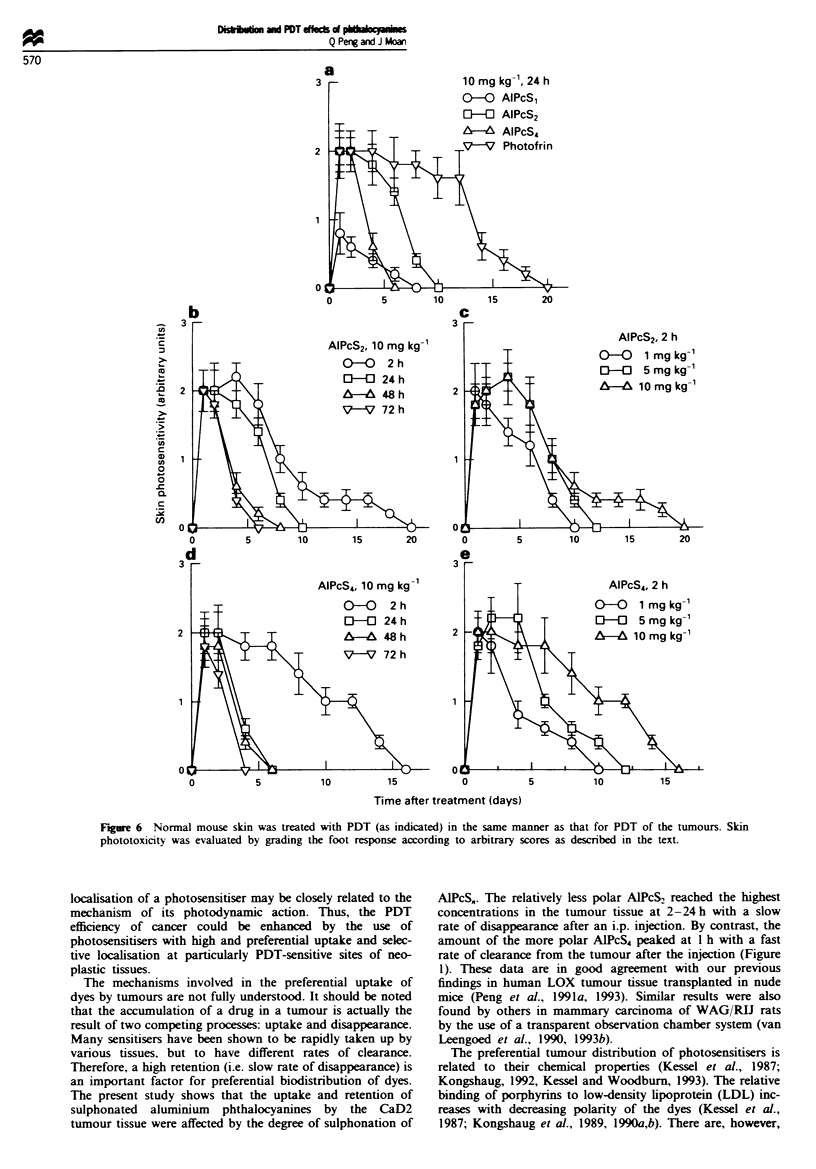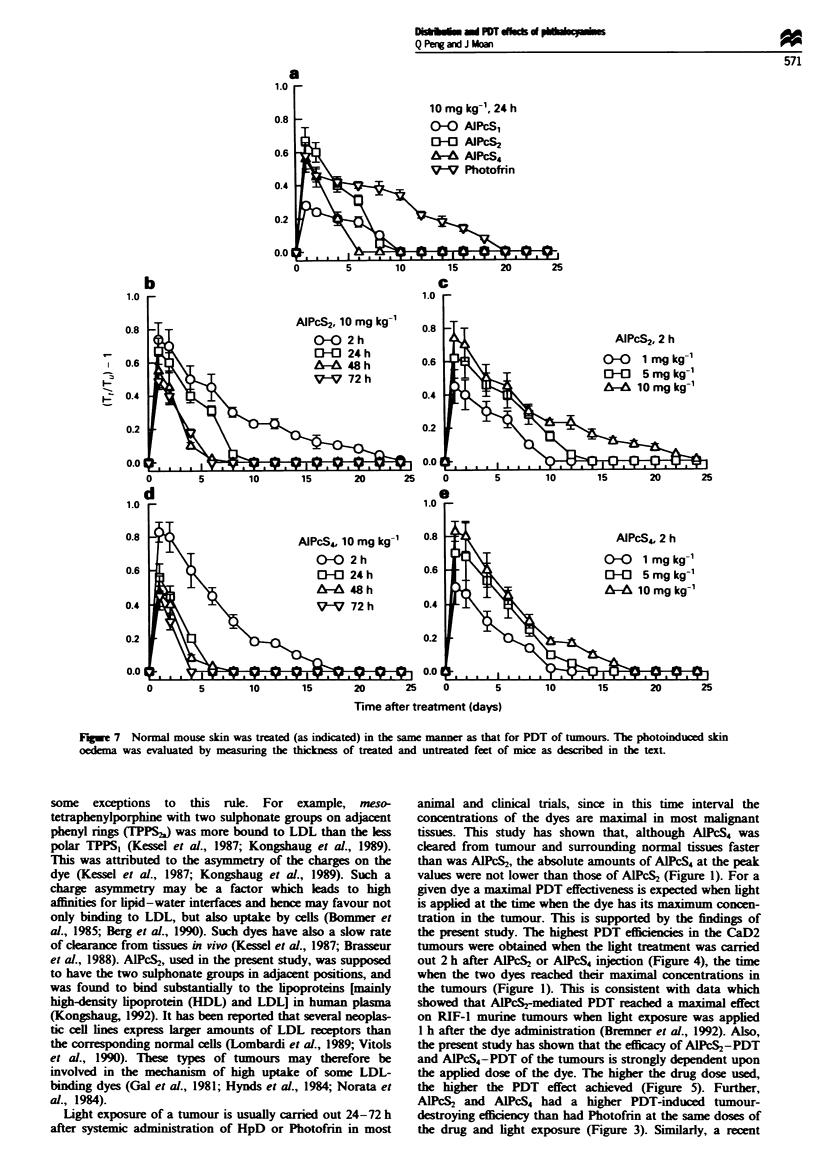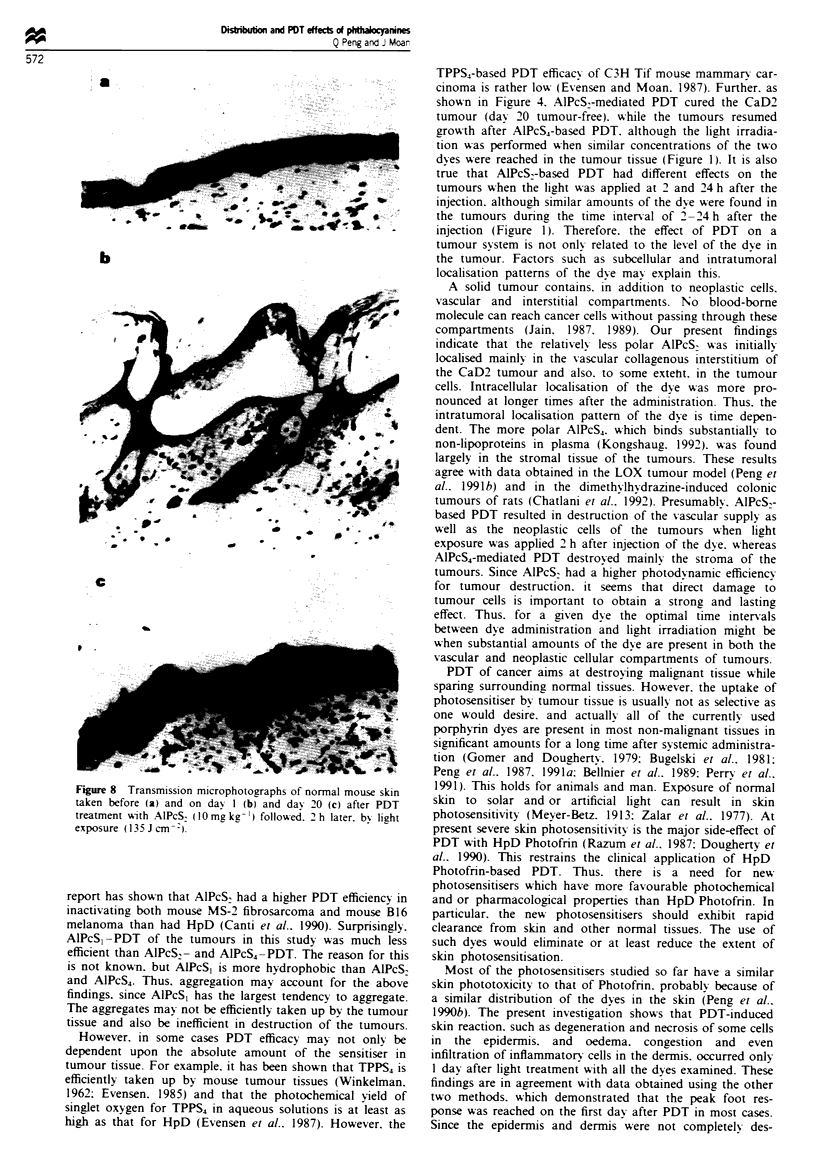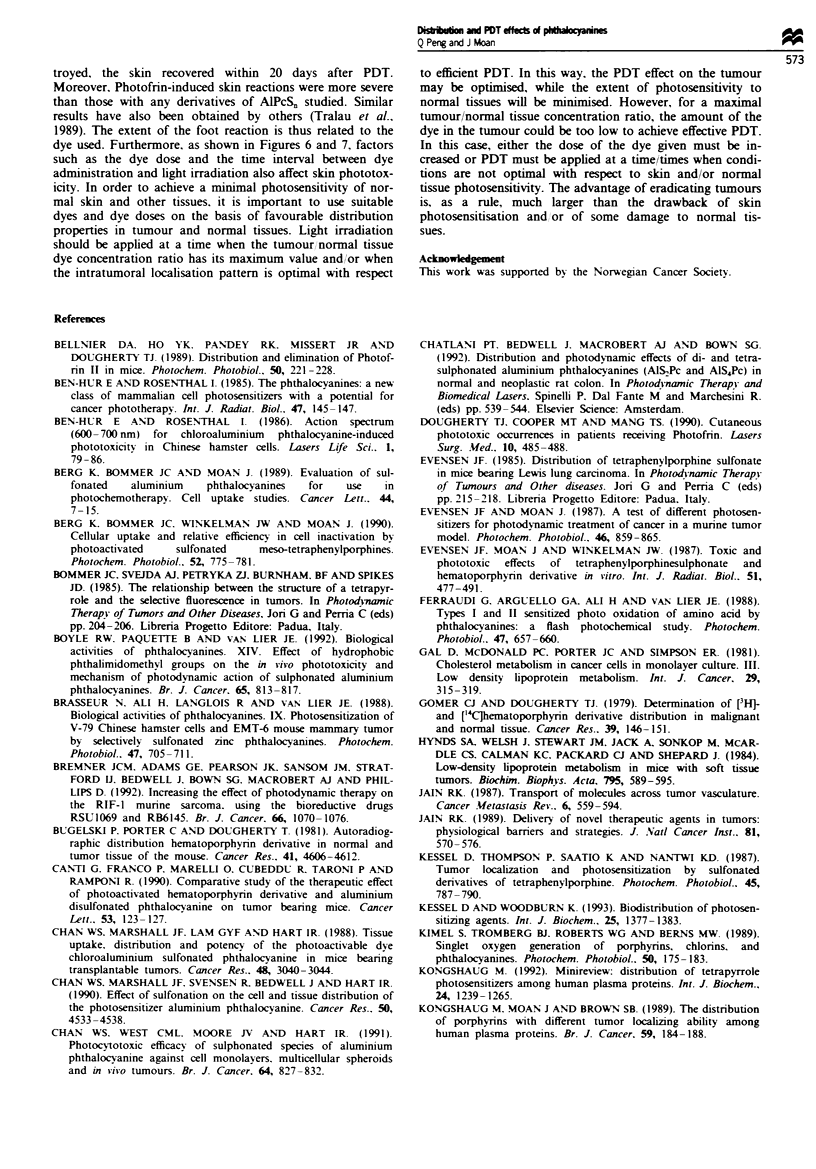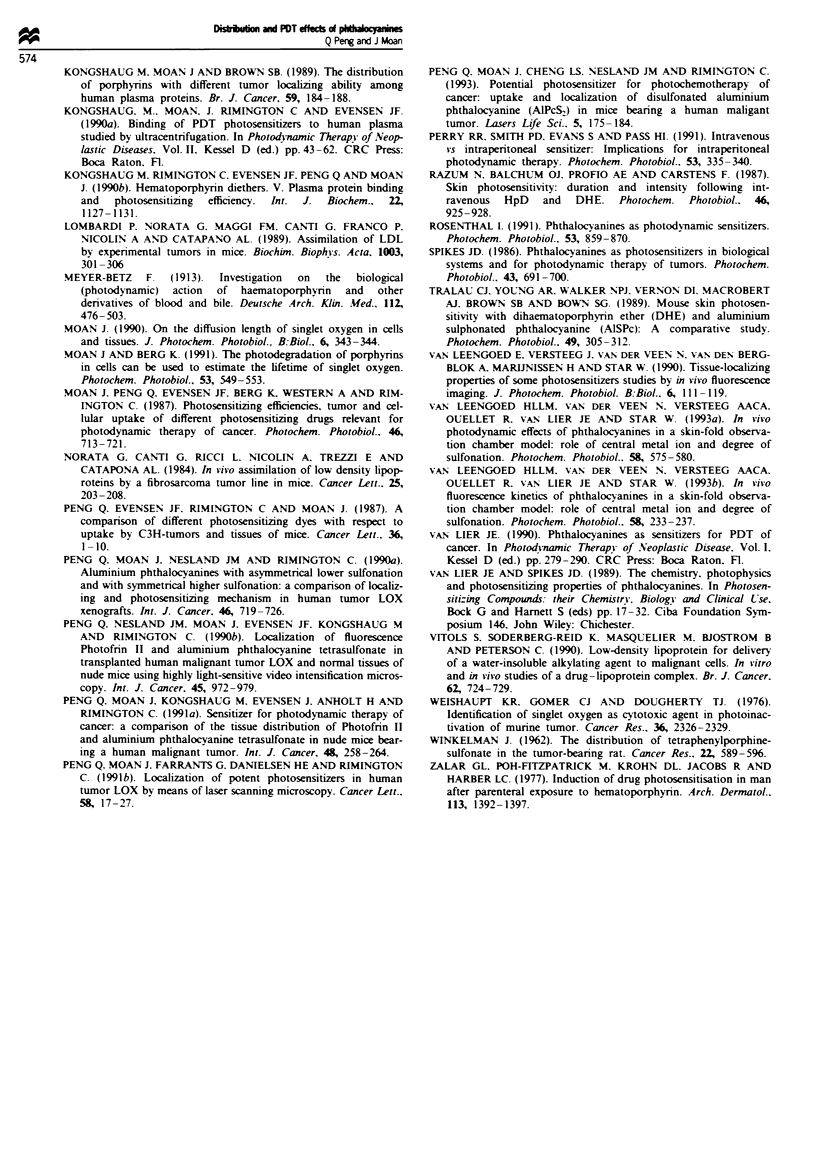# Correlation of distribution of sulphonated aluminium phthalocyanines with their photodynamic effect in tumour and skin of mice bearing CaD2 mammary carcinoma.

**DOI:** 10.1038/bjc.1995.375

**Published:** 1995-09

**Authors:** Q. Peng, J. Moan

**Affiliations:** Department of Pathology, Norwegian Radium Hospital, Montebello, Oslo.

## Abstract

**Images:**


British Journal of Cancer (1995) 72, 565-574

© 1995 Stockton Press All rights reserved 0007-0920/95 $12.00

### Correlation of distribution of sulphonated aluminium phthalocyanines with their photodynamic effect in tumour and skin of mice bearing CaD2 mammary carcinoma

Q Peng[1,2] and J Moan[2]

*Departments of [1]Pathology and [2]Biophysics, Institute for Cancer Research, The Norwegian Radium Hospital, Montebello, 0310 Oslo, Norway.*

**Summary** A chemical extraction assay and fluorescence microscopy incorporating a light-sensitive thermoelectrically cooled charge-coupled device (CCD) camera was used to study the kinetics of uptake, retention and localisation of disulphonated aluminium phthalocyanine ($AlPcS_2$) and tetrasulphonated aluminium phthalocyanine ($AlPcS_4$) at different time intervals after an i.p. injection at a dose of $10\ mg\ kg^{-1}$ body weight (b.w.) in tumour and surrounding normal skin and muscle of female $C_3D_2F_1$ mice bearing CaD2 mammary carcinoma. Moreover, the photodynamic effect on the tumour and normal skin using sulphonated aluminium phthalocyanines ($AlPcS_1$, $AlPcS_2$, $AlPcS_4$) and Photofrin was compared with respect to dye, dye dose and time interval between dye administration and light exposure. The maximal concentrations of $AlPcS_2$ in the tumour tissue were reached 2-24 h after injection of the dye, while the amounts of $AlPcS_4$ peaked 1-2 h after the dye administration. $AlPcS_2$ was simultaneously localised in the interstitium and in the neoplastic cells of the tumour, whereas $AlPcS_4$ appeared to localise only in the stroma of the tumour. The photodynamic efficiency (light was applied 24 h after dye injection at a dose of $10\ mg\ kg^{-1}$ b.w.) of the tumours was found to decrease in the following order: $AlPcS_2 > AlPcS_4 >$ Photofrin $> AlPcS_1$. Furthermore, photodynamic efficacy was strongly dependent upon dye doses and time intervals between dye administration and light exposure: the higher the dose, the higher the photodynamic efficiency. The most efficient photodynamic therapy (PDT) of the tumour was reached (day 20 tumour-free) when light exposure took place 2 h after injection of $AlPcS_2$ ($10\ mg\ kg^{-1}$). A dual intratumoral localisation pattern of the dye, as found for $AlPcS_2$, seems desirable to obtain a high photodynamic efficiency. The kinetic patterns of uptake, retention and localisation of $AlPcS_2$ and $AlPcS_4$ are roughly correlated with their photodynamic effect on the tumour and normal skin.

**Keywords:** photodynamic therapy; sulphonated aluminium phthalocyanines; Photofrin; mouse CaD2 mammary carcinoma; fluorescence microscopy

Although observations that phthalocyanines had affinity for tumour tissues were documented more than 30 years ago (see references in Rosenthal, 1991), interest in phthalocyanines as second-generation photosensitisers for photodynamic therapy (PDT) of cancer arose in 1985 when Ben-Hur and Rosenthal (1985) reported that some phthalocyanines were efficient photosensitisers in mammalian cells. Phthalocyanines (Pcs) can be regarded as azaporphyrins containing a ring system made up of four isoindoles linked by nitrogen atoms. Several diamagnetic metal ions can be inserted into the central ring of the Pc macrocycle, such as aluminium, gallium, tin and zinc, leading to high triplet yields as well as long triplet lifetimes of some of the metallo-Pcs (M-Pcs). M-Pcs are insoluble in water, but water-soluble M-Pcs can be obtained by sulphonation procedures. At present, most studies on Pcs related to PDT have been conducted with water-soluble sulphonated M-Pcs, in particular sulphonated aluminium phthalocyanines ($AlPcS_n$s). M-Pcs have several advantages over haematoporphyrin derivative (HpD) and Photofrin, the dyes currently most used in clinical trials, such as high chemical stability and a well-defined chemical structure (Spikes, 1986; Rosenthal, 1991). Moreover, M-Pcs have an absorption peak around 650-700 nm (Q-band) besides an ultraviolet peak (350 nm). The extinction coefficient of the Q-band used for PDT is about 50 times higher than that of HpD Photofrin, thus probably allowing a more efficient utilisation of photons. Furthermore, the absorption peak of M-Pcs in the Q-band is red-shifted by about 50 nm compared with those of HpD Photofrin. This results in approximately 50% deeper tissue penetration of the activating light (Ben-Hur and Rosenthal, 1986).

Initially, the majority of these studies employed AlPcS in the form of a mixture containing monosulphonated, disulphonated, trisulphonated and tetrasulphonated components (van Lier and Spikes, 1989; van Lier, 1990). Recently, more detailed studies using $AlPcS_n$s with different degrees of sulphonation have been carried out. It has been shown that the degree of sulphonation of $AlPcS_n$s could significantly affect the distribution and the PDT effect of the dyes in some tumour and normal tissues of mice (Chan *et al.*, 1990, 1991; Peng *et al.*, 1991*a*, 1993; Boyle *et al.*, 1992; van Leengoed *et al.*, 1993*a*).

The phenomenon of preferential distribution (uptake and localisation) of a sensitiser in tumours is a basis for selective eradication of neoplasia by PDT. The concentration of a dye within a tumour varies with time after administration. Also, the intratumoral localisation pattern of the dye in the tumour depends upon time course (Peng *et al.*, 1990*a*; 1991*b*), which may affect PDT efficacy. Thus, the optimal time interval between sensitiser application and its subsequent activation by light is a crucial factor for success of PDT. However, few data exist as to correlation of uptake and localisation of M-Pcs in tumours with their photodynamic effect, although a large number of reports indicate the potential utility of M-Pcs as sensitisers for PDT of tumours (Spikes, 1986; van Lier, 1990; Rosenthal, 1991). In the present work, we have studied uptake, elimination, localisation and photodynamic efficacy of $AlPcS_2$ and $AlPcS_4$ in tumours and normal skin of mice bearing CaD2 mammary carcinoma.

### Materials and methods

### *Chemicals*

Derivatives of aluminium phthalocyanines with mono-, di- and tetrasulphonate groups ($AlPcS_1$, $AlPcS_2$ and $AlPcS_4$) were obtained from Porphyrin Products (Logan, UT, USA). These derivatives were assessed by high-performance liquid chromatography (HPLC) to be >90% pure (Berg *et al.*,

Correspondence: Q Peng, Department of Biophysics
Received 25 January 1995; revised 18 April 1995; accepted 24 April 1995

1989). The dye called $AlPcS_2$ in the present study probably contains two sulphonate groups on adjacent phenyl rings ($AlPcS_{2a}$). Stock solutions of $AlPcS_2$ and $AlPcS_4$ were prepared in Dulbecco's phosphate-buffered saline (PBS) (Gibco), while $AlPcS_1$ was dissolved initially in a small amount of 40% ethanol in PBS followed by dilution in PBS. All solutions of $AlPcS_n$s were sonicated for 5 min (Elma Transsonic, type T400, Germany) before use in order to reduce the degree of aggregation. All chemicals used were of the highest purity commercially available.

*Animals and tumour line*

Female $C_3D_2/F_1$ mice were obtained from Bomholtgaard, Ry, Denmark, housed eight per cage and kept under specific pathogen-free conditions. The mice were 6 weeks old and weighed 20–22 g when the experiments started. The CaD2 mouse mammary carcinoma (German Cancer Center, Heidelberg, Germany) was propagated by serial transplantation into the $C_3D_2/F_1$ mice. Non-necrotic tumour material for inoculation was obtained by sterile dissection of large flank tumours from syngeneic mice. Macroscopically viable tumour tissue was gently minced with a pair of scissors and forced repeatedly through sterile needles of diminishing sizes from 19 gauge to 25 gauge to make a tumour tissue suspension, 0.02 ml of which was then injected into the dorsal side of the right hind foot of each mouse. The rate of successful transplantations was nearly 100% in the present experiments. No spontaneous necrosis was observed in the tumours which grew to reach 5–7 mm transverse diameter on the day of treatment, as measured with a caliper. The tumour volume was calculated using the following formula:

$$V = \pi/6(D_1 \times D_2 \times D_3)$$

where $D_1$, $D_2$ and $D_3$ are three orthogonal diameters of the tumours which were measured daily by the caliper (Evensen and Moan, 1987).

*Uptake and elimination of $AlPcS_2$ and $AlPcS_4$ in tumour and surrounding normal tissues*

When the tumours had reached the appropriate size (as indicated above), the mice were given an i.p. injection of 10 mg $kg^{-1}$ b.w. of either $AlPcS_2$ or $AlPcS_4$. At 0.5, 1, 2, 4, 24, 48, 72, 96 and 120 h (five mice for each time point) after the injection the mice were killed by cervical dislocation. The tumour, normal skin overlying the tumour and adjacent normal thigh muscle were removed for determination of $AlPcS_2$ and $AlPcS_4$. The same tissue samples were also taken from control mice receiving no dye. Extraction of $AlPcS_2/AlPcS_4$ from various tissue samples was carried out according to Chan *et al.* (1988) with slight modification. Briefly, the tissue samples were digested with 0.1 M sodium hydroxide (0.1 g of wet tissue in 5 ml of 0.1 M sodium hydroxide) for 4 h in a 50°C water bath with constant shaking. It was found that such a treatment (i.e. 50°C for 4 h in 0.1 M sodium hydroxide solution) did not alter the fluorescence spectra or the fluorescence intensity of test samples containing $AlPcS_2$ or $AlPcS_4$. The resulting solutions were centrifuged at 3000 r.p.m. (1600 g) for 10 min, after which the drug levels in the supernatant were determined by recording fluorescence emission spectra using a Perkin-Elmer LS-5 luminescence spectrofluorimeter. The excitation wavelength was set at 350 nm for both of the drugs, the emission slit width was 5.0 nm and the emission wavelength was scanned from 550 to 750 nm. A cut-off filter was used to remove scattered light of wavelength shorter than 545 nm from the light reaching the detection system of the spectrometer. The absolute amounts of the dyes in tissues were calculated from standard curves made by addition of known amounts of the dye to corresponding tissue extracts from control mice receiving no injection of the dye, and expressed as μg of $AlPcS_2$ or $AlPcS_4$ $g^{-1}$ wet tissue.

*Localisation of $AlPcS_2$ and $AlPcS_4$ in the tumour and surrounding normal tissues*

In the uptake study the tumour and surrounding normal skin and muscle tissues at 2, 24, 48, 72 and 120 h after injection of either $AlPcS_2$ or $AlPcS_4$ were excised and immediately bisected. One half of each tissue sample was used for the extraction assay and the other half was prepared for the localisation study. The samples were immediately immersed in liquid nitrogen, then mounted in medium (Tissue Tek II embedding compound; BDH, Poole, UK). Sections were cut with a cryostat to a thickness of 8 μm and mounted on clean glass slides. A series of sections were cut from each tissue block. The fluorescence localisation pattern of $AlPcS_2$ or $AlPcS_4$ in each section was directly observed by fluorescence microscopy. The same frozen sections were subsequently stained with haematoxylin and eosin (H&E).

Comparisons were made between the fluorescence images and ordinary micrographs of H&E-stained specimens in order to determine the exact histological localisation of $AlPcS_2$ and $AlPcS_4$ in the tissues. The fluorescence microscopy was carried out using an Axioplan microscope (Zeiss, Germany) with a 100 W mercury lamp. The fluorescence images were recorded by a highly light-sensitive thermoelectrically cooled charge-coupled device (CCD) camera (resolution 385 × 578) (Astromed CCD 3200, Cambridge, UK) and hardcopied on a video printer (Sony multiscan video printer UP-930). The filter combination used for detection of $AlPcS_2/AlPcS_4$ fluorescence consisted of a 365 nm excitation filter, a 395 nm beam splitter and a >600 nm emission filter.

*PDT efficiency of the tumour with $AlPcS_1$, $AlPcS_2$, $AlPcS_4$ or Photofrin*

Mice with tumours of the appropriate size were divided into four groups for each drug: group 1, neither a dye nor light,

**Figure 1** The quantities of (**a**) $AlPcS_2$ and (**b**) $AlPcS_4$ extracted from CaD2 tumours (●), skin (■) and muscle (▲) as a function of time after an i.p. injection at a dose of 10 mg $kg^{-1}$. Bars (s.d.) are based on five individual animals.

only i.p. administration of 0.1 ml of PBS; group 2, light only on the tumour; group 3, mice given an i.p. 10 mg kg$^{-1}$ injection of $AlPcS_1$, $AlPcS_2$ or $AlPcS_4$ without light exposure; group 4, mice given an i.p. injection of one of the $AlPcS_n$ derivatives at different doses (1, 5 and 10 mg kg$^{-1}$). At 2, 24 and 72 h after injection, the tumours were irradiated (various numbers of mice per drug and time point as indicated in the figures). In a separate group mice (ten mice) bearing the same tumour model were given an i.p. injection of 10 mg kg$^{-1}$ b.w. Photofrin. After 24 h (a standard time for animal and clinical studies with the dye) the tumours were exposed to light. Responses of the treated tumours were evaluated as tumour regression/regrowth time. The size of the tumours was measured every day, and when the treated tumours reached a volume five times that of the volume on the day just before light irradiation the mice were sacrificed. The data based on the measurements of tumour volumes from each group were pooled to represent mean tumour growth curves.

The laser light irradiation of the tumours was performed as previously described (Evensen and Moan, 1987). Unanaesthetised mice were fixed in Lucite jigs specially designed for irradiation. The tumour area was exposed to red light from a dicyanomethylane-2-methyl-6-(p-dimethylaminostyryl)-4H-pyran (DCM) dye laser pumped by a 5 W argon ion laser (Spectra Physics, 164). The tuning range was 610–690 nm. The dye laser was tuned at 675 nm for all derivatives of $AlPcS_n$ and at 632 nm for Photofrin, the tuning being controlled by means of a monochromator. The laser beam was defocused by means of a microscopic ocular. The light was delivered at a fluence rate of 150 mW cm$^{-2}$ for 15 min exposures in all cases. The fluence rate of the light on the tumour area was regularly controlled by a calibrated integrating sphere with a photodiode coupled to a digital multimeter (Keithley Instruments, Germany) before and immediately after light illumination.

### *PDT effect on normal skin of mice with $AlPcS_1$, $AlPcS_2$, $AlPcS_4$ or Photofrin*

The normal foot response of $C_3D_2/F_1$ mice bearing no tumour (3–5 mice per group) to PDT was evaluated. These normal mice were treated with PDT using the derivatives of $AlPcS_n$ or Photofrin in the exactly same manner as those bearing tumours. Different doses of the drugs (1, 5 and 10 mg kg$^{-1}$ for derivatives of $AlPcS_n$ and 10 mg kg$^{-1}$ for Photofrin) and time intervals between drug administration and light exposure (2, 24, 48 and 72 h for $AlPcS_n$s and 24 h for Photofrin) were employed. The light was used at the same doses as those for PDT of tumours. The PDT-induced response of right hind feet of mice was compared with that of the unexposed left hind feet of the same mice as follows:

1. The average thickness (PDT-induced oedema) of the treated foot ($T_t$) and of the untreated foot ($T_u$) (Evensen and Moan, 1987) was measured every second day for 24 days; the response was calculated as $(T_t/T_u) - 1$.
2. The foot response was graded every second day according to the following arbitrary score, in which each score was also divided into five subscores (0.2 for each) based on the reaction degree: 0, no difference from normal; 0.2–1, slight swelling and mild erythema; 1.2–2, severe swelling (or with exudation), erythema or slight necrosis; 2.2–3, necrosis and crust.
3. Histopathological observation 1, 5, 10 and 20 days after treatment.

### Results

### *Uptake and retention of $AlPcS_2$ and $AlPcS_4$ in tumour and normal tissues*

The kinetics of uptake and retention of $AlPcS_2$ and $AlPcS_4$ by the CaD2 tumours and surrounding normal skin and muscle tissues is shown in Figure 1. The maximal concentrations of $AlPcS_2$ in the tumours were reached 2–24 h after injection of the dye. After that, the concentrations gradually decreased with time. The amounts of $AlPcS_4$ in the tumours peaked at 1–2 h after the dye administration, after which the concentrations declined at a faster rate than that of $AlPcS_2$. Both of the dyes had a similar kinetic pattern of uptake and elimination in the surrounding normal skin and muscle (Figure 1). The absolute levels of $AlPcS_2$ and $AlPcS_4$ were much lower in the muscle than in the tumour and skin (Figure 1). The concentration ratios of tumour–skin and tumour–muscle at different time intervals after injection of $AlPcS_2$ or $AlPcS_4$ are presented in Table I. The concentrations of $AlPcS_2$ in the tumour were 0.7–2.2 times as high as those in the skin and 2.5–13 times as high as those in the muscle during the period studied. In the case of $AlPcS_4$, the dye was taken up 0.9–1.7 times more in the tumour than in the skin and 2–12 times more in the tumour than in the muscle.

### *Localisation of $AlPcS_2$ and $AlPcS_4$ in the tumour and surrounding normal tissues*

Strong fluorescence of $AlPcS_2$ was seen in the connective tissue and vascular structure of dermis surrounding the CaD2 tumour and also, to some extent, in the neoplastic cells of the tumour tissue as early as 2 h after injection of the dye (Figure 2a). The fluorescence of the intracellularly localised dye in the tumour tissue was strong 24–72 h after the injection (Figure 2b), while almost no fluorescence could be detected 120 h after the injection. Fluorescence of the dye was hardly seen in the epidermis and muscle in the time invervals studied. In the case of $AlPcS_4$, there was a strong fluorescence of the dye in the connective tissue and vessels of the dermis around the tumour 2 h post injection (Figure 2c). At 24–72 h after injection much less fluorescence of the dye was found in the dermis surrounding the tumour. Some fluorescence appeared to localise mainly in the stromal components of the tumours (Figure 2d). No fluorescence of the dye was found in the epidermis and muscle.

### *PDT efficiency of tumours with $AlPcS_1$, $AlPcS_2$, $AlPcS_4$ or Photofrin*

The growth of the tumours exposed to light 24 h after an i.p. administration of $AlPcS_1$, $AlPcS_2$, $AlPcS_4$ or Photofrin at a

**Table I** The concentration ratios of tumour–skin and tumour–muscle in mice bearing CaD2 mammary carcinoma at different times after an i.p. injection of 10 mg kg$^{-1}$ $AlPcS_2$ or $AlPcS_4$

| | *$AlPcS_2$* | | *$AlPcS_4$* | |
|---|---|---|---|---|
| *Time (h)* | *Tumour–skin* | *Tumour–muscle* | *Tumour–skin* | *Tumour–muscle* |
| 0.5 | 1.7 | 4.8 | 0.9 | 2.2 |
| 1 | 1.4 | 4.2 | 1 | 2 |
| 2 | 1.2 | 2.5 | 1.1 | 2.5 |
| 4 | 1.2 | 3.4 | 1.2 | 2.9 |
| 24 | 1.5 | 9.2 | 1.7 | 10 |
| 48 | 2.2 | 13 | 1.3 | 12 |
| 72 | 1.9 | 12 | 1.4 | 12 |
| 96 | 1.4 | 10 | 1.3 | 10 |
| 120 | 0.7 | 8 | 1.2 | 12 |

dose of 10 mg $kg^{-1}$ is shown in Figure 3. The control tumours (neither dye nor light) grew exponentially with a doubling time of about 1.6 days. Laser light given to tumours of mice receiving no injection of the dye had a slight but insignificant effect on the tumour growth. Tumours of mice treated with $AlPcS_1$-PDT grew a little more slowly than did the control tumours. However, tumours of mice given $AlPcS_2$ or $AlPcS_4$ followed by light exposure showed a significant growth delay. Among the dyes studied, $AlPcS_2$ was the most efficient photosensitiser, being significantly more efficient than $AlPcS_4$ and Photofrin. The PDT efficiencies in the tumour model were found to decrease in the following order: $AlPcS_2$ > $AlPcS_4$ > Photofrin > $AlPcS_1$ (Figure 3). It should be noted that tumours treated with Photofrin-PDT approached the same growth rate as that of control tumours (6–10 days after PDT), in agreement with our earlier work (Evensen and Moan, 1987), while the tumours treated with $AlPcS_2$- or $AlPcS_4$-mediated PDT had a reduced growth rate during the whole period of observation. PDT was more efficient when light irradiation was applied at 2 h than at 24 or 72 h post dye injection in both cases of $AlPcS_2$ and $AlPcS_4$ (Figure 4). The tumours treated with $AlPcS_2$ followed, 2 h later, by light exposure did not resume growth during the 20 days examined. Moreover, as can be

**Figure 2** Fluorescence photomicrographs of CaD2 tumours sampled 2 h (**a** and **c**) and 24 h (**b** and **d**) after an i.p. injection of $AlPcS_2$ (**a** and **b**) or $AlPcS_4$ (**c** and **d**) at a dose of 10 mg $kg^{-1}$. (**a**) Strong fluorescence of the dye mainly in the stroma of the tumour. (**b**) Fluorescence in the neoplastic cells of the tumour. (**c**) Fluorescence of the dye in the area of subcutaneous connective tissue surrounding the tumour. (**d**) Fluorescence distribution in the space between individual tumour cells.

**Figure 3** Growth curves for CaD2 tumours of mice given i.p. $10\,mg\,kg^{-1}$ injection of one of the dyes as indicated followed, 24 h later, by laser irradiation (675 nm for $AlPcS_n$s or 632 nm for Photofrin; $135\,J\,cm^{-2}$ for all cases). ●, Control; □, light only; ∇, $AlPcS_1$; **O**, $AlPcS_2$; ◆, $AlPcS_4$; ▲, Photofrin.

**Figure 4** Growth times of CaD2 tumours of mice given i.p. $10\,mg\,kg^{-1}$ of $AlPcS_2$ or $AlPcS_4$ followed, various times later as indicated, by laser irradiation (675 nm, $150\,mW\,cm^{-2}$ for 15 min) (see details in the text). The numbers of mice are shown at the bottom of the columns. 'Cure' means no regrowth of the treated tumours for the 20 days observed. The error limits were less than 15% of the mean values.

seen in Figure 5, the efficacy of $AlPcS_2$–PDT and $AlPcS_4$–PDT is strongly dependent upon the applied dose of the dye. The higher the dose used, the higher the PDT efficacy achieved.

### *PDT effect on normal skin*

As shown in Figures 6 and 7, the normal skin phototoxicity of the dyes at various doses and time intervals between dye administration and light irradiation was compared by measuring the average thickness of feet as well as by grading the foot reaction. Both evaluating methods showed similar patterns of the normal foot response to PDT within 24 days post treatment. Photofrin was the most damaging drug to the feet. All dyes studied reached maximum average scores of 2.0–2.2, except $AlPcS_1$, which had a much lower foot response score of 0.8 after PDT at a dose of $10\,mg\,kg^{-1}$ and a 24 h time interval between dye administration and light exposure. Photofrin-mediated PDT showed no significant decrease in foot photosensitivity until 12 days after PDT and did not completely recover until about 20 days following treatment, whereas the foot response induced by $AlPcS_1$-, $AlPcS_2$- or $AlPcS_4$-based PDT was eliminated by 10 days after treatment (Figure 6a). Moreover, when the light exposure was applied 2 h after the injection, both $AlPcS_2$ and $AlPcS_4$ ($10\,mg\,kg^{-1}$) achieved a maximal score of 2.0 the first day after treatment, and still gave scores of 0.6 and 1.0 respectively even 10 days post-PDT, and did not completely

**Figure 5** Growth times of CaD2 tumours in mice given i.p. various doses of $AlPcS_2$ or $AlPcS_4$ as indicated, followed, 2 h later, by light exposure. Otherwise, all conditions were the same as those described in the legend of Figure 4.

recover until day 20 and day 16 following treatment (Figure 6b and d). However, when the light irradiation was performed at later times (24–72 h) after injection, the foot reaction to PDT disappeared more quickly after treatment (Figure 6b and d). In addition, when the doses of the two dyes were reduced from $10\,mg\,kg^{-1}$ to $1\,mg\,kg^{-1}$ and the light irradiation was still given 2 h after dye administration, the foot responses completely disappeared by 10 days after treatment, although a score of 2.0 was reached the first day post PDT in both cases (Figure 6c and e). These results are in good agreement with those obtained from the thickness measurements of treated and untreated feet (Figure 7).

PDT under various conditions (dye, dye dose and time interval between dye administration and light exposure) damaged the epidermis. However, in most cases, the epidermis was not completely destroyed. Degeneration and necrosis of some superficial cells occurred in the epidermis and there was formation of vesicles in the epidermis and at the junction of the epidermis and the dermis (Figure 8b). Damage to sebaceous glands was not pronounced. Interestingly, there was no irreversible injury to the dermis, although vascular reaction in the dermis was evident, such as oedema, congestion and infiltration of white blood cells. The healing of the damaged epidermis seemed to occur promptly via epithelial regeneration (Figure 8c), and the PDT-mediated vascular reaction in the dermis almost disappeared within 20 days post treatment. These histological findings are consistent with the data obtained by the other two evaluating methods.

### Discussion

PDT of cancer is based on the preferential uptake, retention (defined as the inverse of the rate of disappearance of a dye from a tissue) and localisation of photosensitisers in neoplastic tissue. Thus, the elaboration of rational protocols for PDT of cancer must eventually take into consideration the following factors: (1) the kinetics of uptake and disappearance of a photosensitiser in normal and tumour tissues and (2) the localisation patterns of the photosensitiser in such tissues at given times. In particular, an optimal time interval between drug administration and light irradiation should be chosen so as to reach a maximal PDT therapeutic effect on the tumour as well as optimal selectivity. The reaction of singlet oxygen with target biomolecules is regarded as the principal initiating pathway leading to photodynamic damage (Weishaupt *et al.*, 1976; Moan *et al.*, 1987), although free radicals are also thought to be involved in some cases (Ferraudi *et al.*, 1988; Kimel *et al.*, 1989). Since singlet oxygen diffuses intracellularly only about 20 nm in its lifetime (Moan, 1990; Moan and Berg, 1991), the cellular structures close to high sensitiser concentration and high oxygen concentration will be preferentially damaged by the activating light. Consequently, the pattern of intracellular/intratumoral

**Figure 6** Normal mouse skin was treated with PDT (as indicated) in the same manner as that for PDT of the tumours. Skin phototoxicity was evaluated by grading the foot response according to arbitrary scores as described in the text.

localisation of a photosensitiser may be closely related to the mechanism of its photodynamic action. Thus, the PDT efficiency of cancer could be enhanced by the use of photosensitisers with high and preferential uptake and selective localisation at particularly PDT-sensitive sites of neoplastic tissues.

The mechanisms involved in the preferential uptake of dyes by tumours are not fully understood. It should be noted that the accumulation of a drug in a tumour is actually the result of two competing processes: uptake and disappearance. Many sensitisers have been shown to be rapidly taken up by various tissues, but to have different rates of clearance. Therefore, a high retention (i.e. slow rate of disappearance) is an important factor for preferential biodistribution of dyes. The present study shows that the uptake and retention of sulphonated aluminium phthalocyanines by the CaD2 tumour tissue were affected by the degree of sulphonation of AlPcS$_n$. The relatively less polar AlPcS$_2$ reached the highest concentrations in the tumour tissue at 2–24 h with a slow rate of disappearance after an i.p. injection. By contrast, the amount of the more polar AlPcS$_4$ peaked at 1 h with a fast rate of clearance from the tumour after the injection (Figure 1). These data are in good agreement with our previous findings in human LOX tumour tissue transplanted in nude mice (Peng *et al.*, 1991*a*, 1993). Similar results were also found by others in mammary carcinoma of WAG/RIJ rats by the use of a transparent observation chamber system (van Leengoed *et al.*, 1990, 1993*b*).

The preferential tumour distribution of photosensitisers is related to their chemical properties (Kessel *et al.*, 1987; Kongshaug, 1992, Kessel and Woodburn, 1993). The relative binding of porphyrins to low-density lipoprotein (LDL) increases with decreasing polarity of the dyes (Kessel *et al.*, 1987; Kongshaug *et al.*, 1989, 1990*a*,*b*). There are, however,

**Figure 7** Normal mouse skin was treated (as indicated) in the same manner as that for PDT of tumours. The photoinduced skin oedema was evaluated by measuring the thickness of treated and untreated feet of mice as described in the text.

some exceptions to this rule. For example, *meso*-tetraphenylporphine with two sulphonate groups on adjacent phenyl rings ($TPPS_{2a}$) was more bound to LDL than the less polar $TPPS_1$ (Kessel *et al.*, 1987; Kongshaug *et al.*, 1989). This was attributed to the asymmetry of the charges on the dye (Kessel *et al.*, 1987; Kongshaug *et al.*, 1989). Such a charge asymmetry may be a factor which leads to high affinities for lipid–water interfaces and hence may favour not only binding to LDL, but also uptake by cells (Bommer *et al.*, 1985; Berg *et al.*, 1990). Such dyes have also a slow rate of clearance from tissues *in vivo* (Kessel *et al.*, 1987; Brasseur *et al.*, 1988). $AlPcS_2$, used in the present study, was supposed to have the two sulphonate groups in adjacent positions, and was found to bind substantially to the lipoproteins [mainly high-density lipoprotein (HDL) and LDL] in human plasma (Kongshaug, 1992). It has been reported that several neoplastic cell lines express larger amounts of LDL receptors than the corresponding normal cells (Lombardi *et al.*, 1989; Vitols *et al.*, 1990). These types of tumours may therefore be involved in the mechanism of high uptake of some LDL-binding dyes (Gal *et al.*, 1981; Hynds *et al.*, 1984; Norata *et al.*, 1984).

Light exposure of a tumour is usually carried out 24–72 h after systemic administration of HpD or Photofrin in most animal and clinical trials, since in this time interval the concentrations of the dyes are maximal in most malignant tissues. This study has shown that, although $AlPcS_4$ was cleared from tumour and surrounding normal tissues faster than was $AlPcS_2$, the absolute amounts of $AlPcS_4$ at the peak values were not lower than those of $AlPcS_2$ (Figure 1). For a given dye a maximal PDT effectiveness is expected when light is applied at the time when the dye has its maximum concentration in the tumour. This is supported by the findings of the present study. The highest PDT efficiencies in the CaD2 tumours were obtained when the light treatment was carried out 2 h after $AlPcS_2$ or $AlPcS_4$ injection (Figure 4), the time when the two dyes reached their maximal concentrations in the tumours (Figure 1). This is consistent with data which showed that $AlPcS_2$-mediated PDT reached a maximal effect on RIF-1 murine tumours when light exposure was applied 1 h after the dye administration (Bremner *et al.*, 1992). Also, the present study has shown that the efficacy of $AlPcS_2$–PDT and $AlPcS_4$–PDT of the tumours is strongly dependent upon the applied dose of the dye. The higher the drug dose used, the higher the PDT effect achieved (Figure 5). Further, $AlPcS_2$ and $AlPcS_4$ had a higher PDT-induced tumour-destroying efficiency than had Photofrin at the same doses of the drug and light exposure (Figure 3). Similarly, a recent

**Figure 8** Transmission microphotographs of normal mouse skin taken before (**a**) and on day 1 (**b**) and day 20 (**c**) after PDT treatment with $AlPcS_2$ (10 mg $kg^{-1}$) followed, 2 h later, by light exposure (135 J $cm^{-2}$).

report has shown that $AlPcS_2$ had a higher PDT efficiency in inactivating both mouse MS-2 fibrosarcoma and mouse B16 melanoma than had HpD (Canti *et al.*, 1990). Surprisingly, $AlPcS_1$-PDT of the tumours in this study was much less efficient than $AlPcS_2$- and $AlPcS_4$-PDT. The reason for this is not known, but $AlPcS_1$ is more hydrophobic than $AlPcS_2$ and $AlPcS_4$. Thus, aggregation may account for the above findings, since $AlPcS_1$ has the largest tendency to aggregate. The aggregates may not be efficiently taken up by the tumour tissue and also be inefficient in destruction of the tumours.

However, in some cases PDT efficacy may not only be dependent upon the absolute amount of the sensitiser in tumour tissue. For example, it has been shown that $TPPS_4$ is efficiently taken up by mouse tumour tissues (Winkelman, 1962; Evensen, 1985) and that the photochemical yield of singlet oxygen for $TPPS_4$ in aqueous solutions is at least as high as that for HpD (Evensen *et al.*, 1987). However, the $TPPS_4$-based PDT efficacy of C3H Tif mouse mammary carcinoma is rather low (Evensen and Moan, 1987). Further, as shown in Figure 4, $AlPcS_2$-mediated PDT cured the CaD2 tumour (day 20 tumour-free), while the tumours resumed growth after $AlPcS_4$-based PDT, although the light irradiation was performed when similar concentrations of the two dyes were reached in the tumour tissue (Figure 1). It is also true that $AlPcS_2$-based PDT had different effects on the tumours when the light was applied at 2 and 24 h after the injection, although similar amounts of the dye were found in the tumours during the time interval of 2–24 h after the injection (Figure 1). Therefore, the effect of PDT on a tumour system is not only related to the level of the dye in the tumour. Factors such as subcellular and intratumoral localisation patterns of the dye may explain this.

A solid tumour contains, in addition to neoplastic cells, vascular and interstitial compartments. No blood-borne molecule can reach cancer cells without passing through these compartments (Jain, 1987, 1989). Our present findings indicate that the relatively less polar $AlPcS_2$ was initially localised mainly in the vascular collagenous interstitium of the CaD2 tumour and also, to some extent, in the tumour cells. Intracellular localisation of the dye was more pronounced at longer times after the administration. Thus, the intratumoral localisation pattern of the dye is time dependent. The more polar $AlPcS_4$, which binds substantially to non-lipoproteins in plasma (Kongshaug, 1992), was found largely in the stromal tissue of the tumours. These results agree with data obtained in the LOX tumour model (Peng *et al.*, 1991*b*) and in the dimethylhydrazine-induced colonic tumours of rats (Chatlani *et al.*, 1992). Presumably, $AlPcS_2$-based PDT resulted in destruction of the vascular supply as well as the neoplastic cells of the tumours when light exposure was applied 2 h after injection of the dye, whereas $AlPcS_4$-mediated PDT destroyed mainly the stroma of the tumours. Since $AlPcS_2$ had a higher photodynamic efficiency for tumour destruction, it seems that direct damage to tumour cells is important to obtain a strong and lasting effect. Thus, for a given dye the optimal time intervals between dye administration and light irradiation might be when substantial amounts of the dye are present in both the vascular and neoplastic cellular compartments of tumours.

PDT of cancer aims at destroying malignant tissue while sparing surrounding normal tissues. However, the uptake of photosensitiser by tumour tissue is usually not as selective as one would desire, and actually all of the currently used porphyrin dyes are present in most non-malignant tissues in significant amounts for a long time after systemic administration (Gomer and Dougherty, 1979; Bugelski *et al.*, 1981; Peng *et al.*, 1987, 1991*a*; Bellnier *et al.*, 1989; Perry *et al.*, 1991). This holds for animals and man. Exposure of normal skin to solar and/or artificial light can result in skin photosensitivity (Meyer-Betz, 1913; Zalar *et al.*, 1977). At present severe skin photosensitivity is the major side-effect of PDT with HpD/Photofrin (Razum *et al.*, 1987; Dougherty *et al.*, 1990). This restrains the clinical application of HpD/Photofrin-based PDT. Thus, there is a need for new photosensitisers which have more favourable photochemical and/or pharmacological properties than HpD/Photofrin. In particular, the new photosensitisers should exhibit rapid clearance from skin and other normal tissues. The use of such dyes would eliminate or at least reduce the extent of skin photosensitisation.

Most of the photosensitisers studied so far have a similar skin phototoxicity to that of Photofrin, probably because of a similar distribution of the dyes in the skin (Peng *et al.*, 1990*b*). The present investigation shows that PDT-induced skin reaction, such as degeneration and necrosis of some cells in the epidermis, and oedema, congestion and even infiltration of inflammatory cells in the dermis, occurred only 1 day after light treatment with all the dyes examined. These findings are in agreement with data obtained using the other two methods, which demonstrated that the peak foot response was reached on the first day after PDT in most cases. Since the epidermis and dermis were not completely destroyed, the skin recovered within 20 days after PDT. Moreover, Photofrin-induced skin reactions were more severe than those with any derivatives of $AlPcS_n$ studied. Similar results have also been obtained by others (Tralau *et al.*, 1989). The extent of the foot reaction is thus related to the dye used. Furthermore, as shown in Figures 6 and 7, factors such as the dye dose and the time interval between dye administration and light irradiation also affect skin phototoxicity. In order to achieve a minimal photosensitivity of normal skin and other tissues, it is important to use suitable dyes and dye doses on the basis of favourable distribution properties in tumour and normal tissues. Light irradiation should be applied at a time when the tumour/normal tissue dye concentration ratio has its maximum value and/or when the intratumoral localisation pattern is optimal with respect to efficient PDT. In this way, the PDT effect on the tumour may be optimised, while the extent of photosensitivity to normal tissues will be minimised. However, for a maximal tumour/normal tissue concentration ratio, the amount of the dye in the tumour could be too low to achieve effective PDT. In this case, either the dose of the dye given must be increased or PDT must be applied at a time/times when conditions are not optimal with respect to skin and/or normal tissue photosensitivity. The advantage of eradicating tumours is, as a rule, much larger than the drawback of skin photosensitisation and/or of some damage to normal tissues.

**Acknowledgement**
This work was supported by the Norwegian Cancer Society.

**References**

BELLNIER DA, HO YK, PANDEY RK, MISSERT JR AND DOUGHERTY TJ. (1989). Distribution and elimination of Photofrin II in mice. *Photochem. Photobiol.*, **50**, 221–228.

BEN-HUR E AND ROSENTHAL I. (1985). The phthalocyanines: a new class of mammalian cell photosensitizers with a potential for cancer phototherapy. *Int. J. Radiat. Biol.*, **47**, 145–147.

BEN-HUR E AND ROSENTHAL I. (1986). Action spectrum (600–700 nm) for chloroaluminium phthalocyanine-induced phototoxicity in Chinese hamster cells. *Lasers Life Sci.*, **1**, 79–86.

BERG K, BOMMER JC AND MOAN J. (1989). Evaluation of sulfonated aluminium phthalocyanines for use in photochemotherapy. Cell uptake studies. *Cancer Lett.*, **44**, 7–15.

BERG K, BOMMER JC, WINKELMAN JW AND MOAN J. (1990). Cellular uptake and relative efficiency in cell inactivation by photoactivated sulfonated meso-tetraphenylporphines. *Photochem. Photobiol.*, **52**, 775–781.

BOMMER JC, SVEJDA AJ, PETRYKA ZJ, BURNHAM, BF AND SPIKES JD. (1985). The relationship between the structure of a tetrapyrrole and the selective fluorescence in tumors. In *Photodynamic Therapy of Tumors and Other Diseases*, Jori G and Perria C (eds) pp. 204–206. Libreria Progetto Editore: Padua, Italy.

BOYLE RW, PAQUETTE B AND VAN LIER JE. (1992). Biological activities of phthalocyanines. XIV. Effect of hydrophobic phthalimidomethyl groups on the *in vivo* phototoxicity and mechanism of photodynamic action of sulphonated aluminium phthalocyanines. *Br. J. Cancer*, **65**, 813–817.

BRASSEUR N, ALI H, LANGLOIS R AND VAN LIER JE. (1988). Biological activities of phthalocyanines. IX. Photosensitization of V-79 Chinese hamster cells and EMT-6 mouse mammary tumor by selectively sulfonated zinc phthalocyanines. *Photochem. Photobiol.*, **47**, 705–711.

BREMNER JCM, ADAMS GE, PEARSON JK, SANSOM JM, STRATFORD IJ, BEDWELL J, BOWN SG, MACROBERT AJ AND PHILLIPS D. (1992). Increasing the effect of photodynamic therapy on the RIF-1 murine sarcoma, using the bioreductive drugs RSU1069 and RB6145. *Br. J. Cancer*, **66**, 1070–1076.

BUGELSKI P, PORTER C AND DOUGHERTY T. (1981). Autoradiographic distribution hematoporphyrin derivative in normal and tumor tissue of the mouse. *Cancer Res.*, **41**, 4606–4612.

CANTI G, FRANCO P, MARELLI O, CUBEDDU R, TARONI P AND RAMPONI R. (1990). Comparative study of the therapeutic effect of photoactivated hematoporphyrin derivative and aluminium disulfonated phthalocyanine on tumor bearing mice. *Cancer Lett.*, **53**, 123–127.

CHAN WS, MARSHALL JF, LAM GYF AND HART IR. (1988). Tissue uptake, distribution and potency of the photoactivable dye chloroaluminium sulfonated phthalocyanine in mice bearing transplantable tumors. *Cancer Res.*, **48**, 3040–3044.

CHAN WS, MARSHALL JF, SVENSEN R, BEDWELL J AND HART IR. (1990). Effect of sulfonation on the cell and tissue distribution of the photosensitizer aluminium phthalocyanine. *Cancer Res.*, **50**, 4533–4538.

CHAN WS, WEST CML, MOORE JV AND HART IR. (1991). Photocytotoxic efficacy of sulphonated species of aluminium phthalocyanine against cell monolayers, multicellular spheroids and *in vivo* tumours. *Br. J. Cancer*, **64**, 827–832.

CHATLANI PT, BEDWELL J, MACROBERT AJ AND BOWN SG. (1992). Distribution and photodynamic effects of di- and tetrasulphonated aluminium phthalocyanines ($AlS_2Pc$ and $AlS_4Pc$) in normal and neoplastic rat colon. In *Photodynamic Therapy and Biomedical Lasers*, Spinelli P, Dal Fante M and Marchesini R. (eds) pp. 539–544. Elsevier Science: Amsterdam.

DOUGHERTY TJ, COOPER MT AND MANG TS. (1990). Cutaneous phototoxic occurrences in patients receiving Photofrin. *Lasers Surg. Med.*, **10**, 485–488.

EVENSEN JF. (1985). Distribution of tetraphenylporphine sulfonate in mice bearing Lewis lung carcinoma. In *Photodynamic Therapy of Tumours and Other diseases*. Jori G and Perria C (eds) pp. 215–218. Libreria Progetto Editore: Padua, Italy.

EVENSEN JF AND MOAN J. (1987). A test of different photosensitizers for photodynamic treatment of cancer in a murine tumor model. *Photochem. Photobiol.*, **46**, 859–865.

EVENSEN JF, MOAN J AND WINKELMAN JW. (1987). Toxic and phototoxic effects of tetraphenylporphinesulphonate and hematoporphyrin derivative *in vitro*. *Int. J. Radiat. Biol.*, **51**, 477–491.

FERRAUDI G, ARGUELLO GA, ALI H AND VAN LIER JE. (1988). Types I and II sensitized photo oxidation of amino acid by phthalocyanines: a flash photochemical study. *Photochem. Photobiol.*, **47**, 657–660.

GAL D, McDONALD PC, PORTER JC AND SIMPSON ER. (1981). Cholesterol metabolism in cancer cells in monolayer culture. III. Low density lipoprotein metabolism. *Int. J. Cancer*, **29**, 315–319.

GOMER CJ AND DOUGHERTY TJ. (1979). Determination of [$^3$H]- and [$^{14}$C]hematoporphyrin derivative distribution in malignant and normal tissue. *Cancer Res.*, **39**, 146–151.

HYNDS SA, WELSH J, STEWART JM, JACK A, SONKOP M, McARDLE CS, CALMAN KC, PACKARD CJ AND SHEPARD J. (1984). Low-density lipoprotein metabolism in mice with soft tissue tumors. *Biochim. Biophys. Acta*, **795**, 589–595.

JAIN RK. (1987). Transport of molecules across tumor vasculature. *Cancer Metastasis Rev.*, **6**, 559–594.

JAIN RK. (1989). Delivery of novel therapeutic agents in tumors: physiological barriers and strategies. *J. Natl Cancer Inst.*, **81**, 570–576.

KESSEL D, THOMPSON P, SAATIO K AND NANTWI KD. (1987). Tumor localization and photosensitization by sulfonated derivatives of tetraphenylporphine. *Photochem. Photobiol.*, **45**, 787–790.

KESSEL D AND WOODBURN K. (1993). Biodistribution of photosensitizing agents. *Int. J. Biochem.*, **25**, 1377–1383.

KIMEL S, TROMBERG BJ, ROBERTS WG AND BERNS MW. (1989). Singlet oxygen generation of porphyrins, chlorins, and phthalocyanines. *Photochem. Photobiol.*, **50**, 175–183.

KONGSHAUG M. (1992). Minireview: distribution of tetrapyrrole photosensitizers among human plasma proteins. *Int. J. Biochem.*, **24**, 1239–1265.

KONGSHAUG M, MOAN J AND BROWN SB. (1989). The distribution of porphyrins with different tumor localizing ability among human plasma proteins. *Br. J. Cancer*, **59**, 184–188.

KONGSHAUG M. MOAN J AND BROWN SB. (1989). The distribution of porphyrins with different tumor localizing ability among human plasma proteins. *Br. J. Cancer*, **59**, 184-188.

KONGSHAUG, M., MOAN, J. RIMINGTON C AND EVENSEN JF. (1990*a*). Binding of PDT photosensitizers to human plasma studied by ultracentrifugation. In *Photodynamic Therapy of Neoplastic Diseases*, Vol. II. Kessel D (ed.) pp. 43-62. CRC Press: Boca Raton, Fl.

KONGSHAUG M. RIMINGTON C. EVENSEN JF. PENG Q AND MOAN J. (1990*b*). Hematoporphyrin diethers. V. Plasma protein binding and photosensitizing efficiency. *Int. J. Biochem.*, **22**, 1127-1131.

LOMBARDI P. NORATA G. MAGGI FM. CANTI G. FRANCO P. NICOLIN A AND CATAPANO AL. (1989). Assimilation of LDL by experimental tumors in mice. *Biochim. Biophys. Acta*, **1003**, 301-306

MEYER-BETZ F. (1913). Investigation on the biological (photodynamic) action of haematoporphyrin and other derivatives of blood and bile. *Deutsche Arch. Klin. Med.*, **112**, 476-503.

MOAN J. (1990). On the diffusion length of singlet oxygen in cells and tissues. *J. Photochem. Photobiol., B:Biol.*, **6**, 343-344.

MOAN J AND BERG K. (1991). The photodegradation of porphyrins in cells can be used to estimate the lifetime of singlet oxygen. *Photochem. Photobiol.*, **53**, 549-553.

MOAN J. PENG Q. EVENSEN JF. BERG K. WESTERN A AND RIMINGTON C. (1987). Photosensitizing efficiencies, tumor and cellular uptake of different photosensitizing drugs relevant for photodynamic therapy of cancer. *Photochem. Photobiol.*, **46**, 713-721.

NORATA G. CANTI G. RICCI L. NICOLIN A. TREZZI E AND CATAPONA AL. (1984). *In vivo* assimilation of low density lipoproteins by a fibrosarcoma tumor line in mice. *Cancer Lett.*, **25**, 203-208.

PENG Q. EVENSEN JF. RIMINGTON C AND MOAN J. (1987). A comparison of different photosensitizing dyes with respect to uptake by C3H-tumors and tissues of mice. *Cancer Lett.*, **36**, 1-10.

PENG Q. MOAN J. NESLAND JM AND RIMINGTON C. (1990*a*). Aluminium phthalocyanines with asymmetrical lower sulfonation and with symmetrical higher sulfonation: a comparison of localizing and photosensitizing mechanism in human tumor LOX xenografts. *Int. J. Cancer*, **46**, 719-726.

PENG Q. NESLAND JM. MOAN J. EVENSEN JF. KONGSHAUG M AND RIMINGTON C. (1990*b*). Localization of fluorescence Photofrin II and aluminium phthalocyanine tetrasulfonate in transplanted human malignant tumor LOX and normal tissues of nude mice using highly light-sensitive video intensification microscopy. *Int. J. Cancer*, **45**, 972-979.

PENG Q. MOAN J. KONGSHAUG M. EVENSEN J. ANHOLT H AND RIMINGTON C. (1991*a*). Sensitizer for photodynamic therapy of cancer: a comparison of the tissue distribution of Photofrin II and aluminium phthalocyanine tetrasulfonate in nude mice bearing a human malignant tumor. *Int. J. Cancer*, **48**, 258-264.

PENG Q. MOAN J. FARRANTS G. DANIELSEN HE AND RIMINGTON C. (1991*b*). Localization of potent photosensitizers in human tumor LOX by means of laser scanning microscopy. *Cancer Lett.*, **58**, 17-27.

PENG Q. MOAN J. CHENG LS. NESLAND JM AND RIMINGTON C. (1993). Potential photosensitizer for photochemotherapy of cancer: uptake and localization of disulfonated aluminium phthalocyanine ($AlPcS_2$) in mice bearing a human maligant tumor. *Lasers Life Sci.*, **5**, 175-184.

PERRY RR. SMITH PD. EVANS S AND PASS HI. (1991). Intravenous *vs* intraperitoneal sensitizer: Implications for intraperitoneal photodynamic therapy. *Photochem. Photobiol.*, **53**, 335-340.

RAZUM N. BALCHUM OJ. PROFIO AE AND CARSTENS F. (1987). Skin photosensitivity: duration and intensity following intravenous HpD and DHE. *Photochem. Photobiol.*, **46**, 925-928.

ROSENTHAL I. (1991). Phthalocyanines as photodynamic sensitizers. *Photochem. Photobiol.*, **53**, 859-870.

SPIKES JD. (1986). Phthalocyanines as photosensitizers in biological systems and for photodynamic therapy of tumors. *Photochem. Photobiol.*, **43**, 691-700.

TRALAU CJ. YOUNG AR. WALKER NPJ. VERNON DI. MACROBERT AJ. BROWN SB AND BOWN SG. (1989). Mouse skin photosensitivity with dihaematoporphyrin ether (DHE) and aluminium sulphonated phthalocyanine (AlSPc): A comparative study. *Photochem. Photobiol.*, **49**, 305-312.

VAN LEENGOED E. VERSTEEG J. VAN DER VEEN N. VAN DEN BERG-BLOK A. MARIJNISSEN H AND STAR W. (1990). Tissue-localizing properties of some photosensitizers studies by *in vivo* fluorescence imaging. *J. Photochem. Photobiol. B:Biol.*, **6**, 111-119.

VAN LEENGOED HLLM. VAN DER VEEN N. VERSTEEG AACA. OUELLET R. VAN LIER JE AND STAR W. (1993*a*). *In vivo* photodynamic effects of phthalocyanines in a skin-fold observation chamber model: role of central metal ion and degree of sulfonation. *Photochem. Photobiol.*, **58**, 575-580.

VAN LEENGOED HLLM. VAN DER VEEN N. VERSTEEG AACA. OUELLET R. VAN LIER JE AND STAR W. (1993*b*). *In vivo* fluorescence kinetics of phthalocyanines in a skin-fold observation chamber model: role of central metal ion and degree of sulfonation. *Photochem. Photobiol.*, **58**, 233-237.

VAN LIER JE. (1990). Phthalocyanines as sensitizers for PDT of cancer. In *Photodynamic Therapy of Neoplastic Disease*, Vol. I. Kessel D (ed.) pp. 279-290. CRC Press: Boca Raton, Fl.

VAN LIER JE AND SPIKES JD. (1989). The chemistry, photophysics and photosensitizing properties of phthalocyanines. In *Photosensitizing Compounds: their Chemistry, Biology and Clinical Use*, Bock G and Harnett S (eds) pp. 17-32. Ciba Foundation Symposium 146. John Wiley: Chichester.

VITOLS S. SODERBERG-REID K. MASQUELIER M. BJOSTROM B AND PETERSON C. (1990). Low-density lipoprotein for delivery of a water-insoluble alkylating agent to malignant cells. *In vitro* and *in vivo* studies of a drug-lipoprotein complex. *Br. J. Cancer*, **62**, 724-729.

WEISHAUPT KR. GOMER CJ AND DOUGHERTY TJ. (1976). Identification of singlet oxygen as cytotoxic agent in photoinactivation of murine tumor. *Cancer Res.*, **36**, 2326-2329.

WINKELMAN J. (1962). The distribution of tetraphenylporphinesulfonate in the tumor-bearing rat. *Cancer Res.*, **22**, 589-596.

ZALAR GL. POH-FITZPATRICK M. KROHN DL. JACOBS R AND HARBER LC. (1977). Induction of drug photosensitisation in man after parenteral exposure to hematoporphyrin. *Arch. Dermatol.*, **113**, 1392-1397.